# Evaluation of cardiovascular biomarkers in a randomized trial of fosamprenavir/ritonavir vs. efavirenz with abacavir/lamivudine in underrepresented, antiretroviral-naïve, HIV-infected patients (SUPPORT): 96-week results

**DOI:** 10.1186/1471-2334-13-269

**Published:** 2013-06-07

**Authors:** Princy Kumar, Edwin DeJesus, Gregory Huhn, Louis Sloan, Catherine Butkus Small, Howard Edelstein, Franco Felizarta, Ritche Hao, Lisa Ross, Britt Stancil, Keith Pappa, Belinda Ha

**Affiliations:** 1 Georgetown University Hospital, 3800 Reservoir Rd, NW 5PHC Building, Washington, DC, 20007, USA; 2 Orlando Immunology Center, Orlando, FL, USA; 3 Ruth M Rothstein CORE Center, Chicago, IL, USA; 4 Baylor University Medical Center, Dallas, TX, USA; 5 New York Medical College, Valhalla, NY, USA; 6 Alameda County Medical Center, Oakland, CA, USA; 7 Private Practice, Bakersfield, CA, USA; 8 Chase Brexton Health Services, Inc, Baltimore, MD, USA; 9 ViiV Healthcare, Research Triangle Park, Durham, NC, USA; 10 GlaxoSmithKline, Research Triangle Park, Durham, NC, USA

**Keywords:** Abacavir, Cardiovascular biomarker, Efavirenz, Fosamprenavir, HIV, Lamivudine, Minority, Ritonavir, Underrepresented

## Abstract

**Background:**

Rates of cardiovascular disease are higher among HIV-infected patients as a result of the complex interplay between traditional risk factors, HIV-related inflammatory and immunologic changes, and effects of antiretroviral therapy (ART). This study prospectively evaluated changes in cardiovascular biomarkers in an underrepresented, racially diverse, HIV-1-infected population receiving abacavir/lamivudine as backbone therapy.

**Methods:**

This 96-week, open-label, randomized, multicenter study compared once-daily fosamprenavir/ritonavir 1400/100 mg and efavirenz 600 mg, both with ABC/3TC 600 mg/300 mg, in antiretroviral-naïve, HLA-B*5701-negative adults without major resistance mutations to study drugs. We evaluated changes from baseline to weeks 4, 12, 24, 48, and 96 in interleukin-6 (IL-6), high-sensitivity C-reactive protein (hs-CRP), soluble vascular adhesion molecule-1 (sVCAM-1), d-dimer, plasminogen, and fibrinogen. Biomarker data were log-transformed before analysis, and changes from baseline were described using geometric mean ratios.

**Results:**

This study enrolled 101 patients (51 receiving fosamprenavir/ritonavir; 50 receiving efavirenz): 32% female, 60% African American, and 38% Hispanic/Latino; 66% (67/101) completed 96 weeks on study. At week 96, levels of IL-6, sVCAM-1, d-dimer, fibrinogen, and plasminogen were lower than baseline in both treatment groups, and the decrease was statistically significant for sVCAM-1 (fosamprenavir/ritonavir and efavirenz), d-dimer (fosamprenavir/ritonavir and efavirenz), fibrinogen (efavirenz), and plasminogen (efavirenz). Values of hs-CRP varied over time in both groups, with a significant increase over baseline at Weeks 4 and 24 in the efavirenz group. At week 96, there was no difference between the groups in the percentage of patients with HIV-1 RNA <50 copies/mL (fosamprenavir/ritonavir 63%; efavirenz 66%) by ITT missing-equals-failure analysis. Treatment-related grade 2–4 adverse events were more common with efavirenz (32%) compared with fosamprenavir/ritonavir (20%), and median lipid concentrations increased in both groups over 96 weeks of treatment.

**Conclusions:**

In this study of underrepresented patients, treatment with abacavir/lamivudine combined with either fosamprenavir/ritonavir or efavirenz over 96 weeks, produced stable or declining biomarker levels except for hs-CRP, including significant and favorable decreases in thrombotic activity (reflected by d-dimer) and endothelial activation (reflected by sVCAM-1). Our study adds to the emerging data that some cardiovascular biomarkers are decreased with initiation of ART and control of HIV viremia.

**Trial registration:**

ClinicalTrials.gov identifier NCT00727597

## Background

Cardiovascular disease (CVD) is the leading cause of death among Americans [1], is of particular importance in HIV-infected patients because it may be exacerbated by the chronic inflammation and dyslipidemia seen in this patient population [2,3]. Compared with uninfected controls, rates of myocardial infarction (MI) and sudden cardiac death are higher among patients with HIV [4-6]. These outcomes are the result of a complex interplay between traditional CVD risk factors, HIV-related inflammatory and immunologic changes, and the effects of antiretroviral therapy (ART) [2,3,7].

In the United States, racial and ethnic minorities are disproportionately affected by the HIV/AIDS epidemic. Many of the traditional risk factors for CVD such as hypertension, diabetes, and obesity are higher in the African-American population [8]. Clinical trials demographics often do not reflect the diverse nature of the HIV-positive population in the United States. Given the relatively low rates of CVD endpoints, such as myocardial infarction or stroke, many studies use biomarkers of inflammation, thrombogenesis, and/or endothelial activation to evaluate the effects of HIV and/or ART on the cardiovascular system [2-4,9-16]. Data on the predictive value of individual cardiovascular biomarkers in HIV-infected patients are still emerging [2,3], and, to date, no study has prospectively examined these biomarkers in an exclusively non-white population.

The present study was a randomized trial comparing ritonavir-boosted fosamprenavir (FPV/r) versus EFV, both in combination with abacavir/lamivudine (ABC/3TC), in an ART-naïve, HIV-infected population that is often underrepresented in clinical trials in the United States. The primary endpoint was a measure of tolerability, and the study included a prospective, real-time evaluation of changes of pre-specified cardiovascular biomarkers over 96 weeks. The final, 96-week analysis of the trial with a focus on cardiovascular biomarker data is described in this report.

## Methods

### Study design

This randomized, open-label, multi-centered, pilot study enrolled patients ≥18 years of age who were ART-naïve (≤14 days of treatment with any ART), HLA-B*5701-negative, and had a screening HIV-1 RNA >5000 copies/mL. Patients were required to be of minority race or ethnicity. Females of child-bearing potential could not be pregnant or breastfeeding at screening and had to agree to use a suitable form of contraception, including abstinence, double barrier method, or intrauterine device, throughout the study. Hormonal contraception was not recommended for female patients taking FPV/r because of decreased efficacy of contraception and increased risk of hepatic transaminase elevation.

Patients were excluded if they had an active or acute CDC Clinical Category C event (AIDS-defining illness) within 28 days of screening or had chronic hepatitis B infection (HBsAg+), hepatitis C infection requiring active treatment, clinically-relevant pancreatitis or hepatitis, AST or ALT >5× upper limit of normal, any grade 4 laboratory abnormality, hemoglobin <8 g/dL, platelet count <50,000/mm^3^, or calculated creatinine clearance <50 mL/min via Cockcroft-Gault equation at screening. Use of immunomodulators (e.g., interleukins, interferons, cyclosporine), any vaccinations, systemic cytotoxic chemotherapy, or investigational therapy was prohibited within 28 days of study entry. Patients were also excluded if their screening HIV-1 genotype indicated the presence of specific mutations. In the reverse transcriptase (RT) region, exclusionary mutations were those associated with resistance to abacavir, lamivudine, or efavirenz (K65R, L74V, K103N, Y115F, Y181C/I, Y188C/L/H, G190S/A), or a combination of two or more thymidine analog mutations (M41L, D67N, K70R, K219Q or E) that included changes at either L210 or T215. Within the protease region, exclusionary mutations were those associated with resistance to fosamprenavir or ritonavir (I50V, I54L/M, I84V) or the combination of V32I + I147V. There were no restrictions on screening CD4 cell count.

All patients provided written informed consent to participate in the study, and the protocol was approved by the institutional review board for each study site. Eligible patients were randomized 1:1 to receive either FPV 1400 mg once daily (LEXIVA®, ViiV Healthcare, Research Triangle Park, NC) plus ritonavir 100 mg once daily (Norvir®, Abbott Laboratories, North Chicago, IL) or EFV 600 mg once daily (Sustiva®, Bristol-Myers Squibb, Princeton, NJ). All patients also received ABC 600 mg + 3TC 300 mg once daily (EPZICOM®, ViiV Healthcare, Research Triangle Park, NC). Randomization was stratified by screening HIV-1 RNA <100,000 and ≥100,000 c/mL.

For the virology HIV-1 genotypic analysis, virologic failure was defined as having either virologic non-response (failure to achieve HIV-1 RNA <400 c/mL by Week 24) or confirmed virologic rebound (reduction of HIV RNA to <400 c/mL by Week 24 with a subsequent increase to ≥400 c/mL on two consecutive occasions 2 to 4 weeks apart.) In the case of clinically-suspected hypersensitivity to ABC, patients were permitted to substitute ABC/3TC with another suitable dual nucleoside/nucleotide RT inhibitor combination chosen by the site investigator and remained in the study.

### Measurements

Clinical, safety, and laboratory evaluations were performed at screening, baseline, and at weeks 2, 4, 8, 12, 24, 36, 48, 60, 72, 84, and 96 including measurements of plasma HIV-1 RNA, CD4 cell count, hematology, and blood chemistry. HIV-1 RNA was measured using the standard or ultrasensitive Roche Amplicor HIV-1 Monitor assay (Roche Diagnostics, Branchburg, New Jersey). A fasting lipid panel was done at baseline and every 24 weeks thereafter through week 96. All laboratory tests were performed centrally by Quest Diagnostics (Valencia, California, USA). Adverse events (AEs) and laboratory toxicities were graded using the 2004 Division of AIDS Toxicity Grading Scale.

### Cardiovascular biomarker measurements

Biomarkers were assessed in real-time using whole blood, plasma, or serum samples collected at baseline and at weeks 4, 12, 24, 48, and 96. Six biomarkers were assessed including high-sensitivity C-reactive protein [hs-CRP] and interleukin-6 [IL-6] which are associated with inflammation; d-dimer, plasminogen, and fibrinogen which are associated with thrombogenesis; and sVCAM-1 which is associated with endothelial activation. Biomarkers were assayed centrally by Quest Diagnostics (Valencia, California, USA). sVCAM-11 and IL-6 were analyzed by quantitative sandwich ELISA; plasminogen by double antibody ELISA; fibrinogen by photo-optical method; d-dimer by immunoturbidimetry; and hs-CRP by fixed-time nephelometry. All assays were performed according to the manufacturers’ recommendations and used their normal ranges.

### Endpoints

The pre-specified primary endpoint of this study was the time to switch of comparator drugs (FPV/r or EFV) or time to development of any treatment-related grade 3–4 AE. Pre-specified secondary endpoints included change from baseline in biomarkers of cardiovascular risk; measures of virologic efficacy; incidence, severity, and causality of adverse events and laboratory abnormalities; and change from baseline in CD4 cell count and fasting lipids. Patients experiencing virologic failure were also evaluated for treatment-emergent viral resistance mutations.

### Statistical analysis

A sample size of 100 patients was planned for this pilot study based on practical considerations. Analyses were performed using the intent-to-treat exposed (ITT-E) population that included any enrolled patient who took at least one dose of study medication. Biomarker data was log-transformed prior to analysis (due to the highly skewed nature of the data), and the change from baseline was assessed using geometric mean ratios with 95% confidence intervals.

Efficacy results were assessed using missing or discontinuation equals failure (MD = F) analyses, in which missing assessments were considered failures, and observed analyses, in which missing assessments at any scheduled time point were considered unevaluable and were not imputed. The primary endpoint was compared using the log-rank test between the two treatment groups. Virologic, immunologic, and safety results were described using summary statistics.

## Results

### Patient characteristics and accountability

This study, which enrolled 101 patients (51 on FPV/r; 50 on EFV), ran from July 2008 (first patient first visit) through March 2011 (last patient last visit). Baseline characteristics are shown in Table 1. The number of female patients and the racial/ethnic distribution was similar between the two treatment groups. At screening, the percentage of patients with HIV RNA ≥100,000 c/mL was similar between groups, but more patients in the FPV/r-containing group had CD4 cell counts <200 cells/mm^3^ (41% FPV/r; 34% EFV). Cardiovascular risk factors were generally similar between the two groups. Sixty-six percent of patients completed 96 weeks on study (Table 2). In both groups, the most common reasons for premature discontinuation were lack of efficacy and lost to follow-up.

**Table 1:** Baseline patient demographics, characteristics, and medical history

	**FPV/r + ABC/3TC (n = 51)**	**EFV + ABC/3TC (n = 50)**
Median age, years (range)	34.0 (18–79)	33.5 (19–55)
Female, n (%)	16 (31%)	16 (32%)
Race/ethnicity, n (%)		
African American, non-Hispanic	29 (57%)	33 (66%)
Hispanic, non-African American	18 (35%)	13 (26%)
African American and Hispanic	3 (6%)	4 (8%)
Asian	1 (2%)	0
Median plasma HIV-1 RNA, log_10_ copies/mL	4.96	4.82
≥100,000 copies/mL, n (%)	22 (43%)	20 (40%)
Median CD4 cell count, cells/mm^3^ (range)	237.0 (19–1061)	272.5 (19–699)
<200 cells/mm^3^, n (%)	21 (41%)	17 (34%)
<50 cells/mm^3^, n (%)	3 (6%)	7 (14%)
Positive hepatitis C serology	5 (10%)	5 (10%)
CDC classification for HIV infection, n (%)		
Category A	40 (78%)	43 (86%)
Category B	4 (8%)	7 (14%)
Category C	7 (14%)	0
Framingham risk score <5%*, n (%)	28 (78%)	28 (88%)
Diabetes type II, n (%)	3 (6%)	1 (2%)
Hypercholesterolemia, n (%)	17 (33%)	14 (28%)
Hypertension, n (%)	9 (18%)	12 (24%)
Tobacco use		
Never smoked, n (%)	26 (51%)	19 (38%)
Current smoker, n (%)	20 (39%)	20 (40%)
Former smoker, n (%)	5 (10%)	11 (22%)
Years smoked, median (range)^†^	10 (2–39)	10 (0–33)

*FPV/r, n = 36; EFV, n = 32. Framingham scores were calculated only in non-diabetic individuals aged 30 years or older. ^†^FPV/r, n = 24; EFV, n = 30.

Abbreviations: *ABC/3TC* abacavir/lamivudine, *CDC* Centers for Disease Control and Prevention, *EFV* efavirenz, *FPV/r* fosamprenavir/ritonavir.

**Table 2:** Patient accountability

	**FPV/r + ABC/3TC**	**EFV + ABC/3TC**
ITT:Exposed population, n	51	50
Completed 96 weeks on study, n (%)	34 (67%)	33 (66%)
Discontinued study, n (%)	17 (33%)	17 (34%)
Adverse event	3 (6%)	2 (4%)
Lack of efficacy	5 (10%)	5 (10%)
Non-compliance with protocol treatment	3 (6%)	2 (4%)
Lost to follow-up	6 (12%)	6 (12%)
Withdrew consent	0	2 (4%)

Abbreviations: *ABC/3TC* abacavir/lamivudine, *EFV* efavirenz, *FPV/r* fosamprenavir/ritonavir, *ITT* intent-to-treat.

### Primary endpoint

The primary endpoint of this study was the time to switch of comparator drugs (FPV/r or EFV) or development of any treatment-related grade 3/4 AE. In the FPV/r-containing group, 4/51 (8%) of patients reached the latter endpoint compared with 6/50 (12%) in the EFV-containing group (*P* = 0.656). Of the 4 patients in the FPV/r-containing group, 1 switched from FPV/r to EFV after 14 days due to an adverse event, 1 had grade 3 hypertriglyceridemia at day 57 on treatment, 1 had a fatal cardiac arrest at day 200, and 1 had grade 3 worsening hyperlipidemia at day 340. The cardiac arrest occurred shortly after the Week 24 study visit in a 49-year old Central/South Asian male, non-smoker, with a history of hypercholesterolemia and uncontrolled hypertension; on autopsy, death was attributed to atherosclerotic coronary artery disease resulting from 95% narrowing of the left anterior descending coronary artery by necrotic plaque. In the EFV-containing group, 3 of 6 patients experienced grade 3 AEs (rash at day 13, acute pancreatitis at day 33, and high cholesterol at day 337) and 3 switched from EFV to FPV/r (days 31, 327, and 463) for adverse events. There was no apparent relationship between the primary endpoint and baseline viral load.

### Virologic and immunologic responses

**Figure 1:**
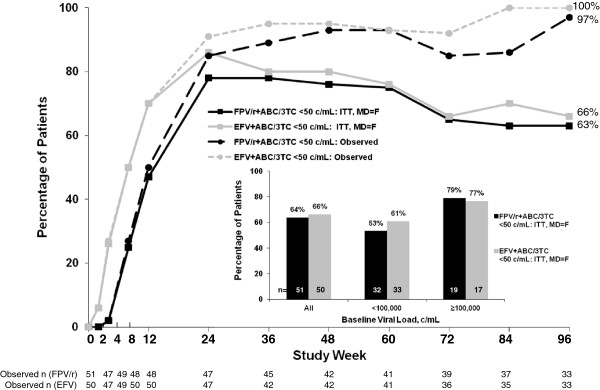
**Virologic response (% of patients with HIV-1 RNA <50 copies/mL) over 96 weeks.** Virologic response by screening HIV-1 RNA.

There was no difference between treatment groups in the percentage of patients who achieved HIV RNA <50 c/mL at Week 96 using either an ITT -:MD = F or an observed analysis (Figure 1). Patients with screening HIV-1 RNA ≥100,000 c/mL had higher rates of viral suppression at Week 96 by ITT -:MD = F than patients with screening HIV-1 RNA <100,000 c/mL (Figure 1 inset). Over the 96-week study, the median change from baseline in CD4 cell count was +186 cells/mm^3^ (interquartile range: 136 to 311 cells/mm^3^) in the FPV/r group and +235 cells/mm^3^ (range: 137 to 294 cells/mm^3^) in the EFV group.

For the virology analysis, by week 24, all on-study subjects had virologically suppressed to <400 copies/ml. Confirmed virologic failure occurred in 7 patients (14%) taking FPV/r and 6 patients (12%) taking EFV. In the FPV/r-containing group, the following treatment-emergent viral mutations in RT were observed at virologic failure: M184V (in virus from 2 patients); while virus from another patient developed a treatment-emergent secondary protease mutation V77I (per IAS-USA guidelines). In the EFV-containing group, the following treatment-emergent viral mutations in RT were observed at virologic failure: M184I/M (in virus from 1 patient), K65R (virus from 1 patient), and K103N (virus from 2 patients). Interestingly, virus from 1 patient experiencing virologic failure while taking EFV also developed several treatment-emergent secondary PI-associated mutations.

### Safety and tolerability

Treatment-related serious AEs occurred in 2 patients taking FPV/r (cardiac arrest and suspected drug hypersensitivity to ABC) and in 3 patients taking EFV (acute pancreatitis, depression, and suspected drug hypersensitivity to ABC). Through Week 96, 20% (10/51) of patients taking FPV/r and 32% (16/50) taking EFV experienced treatment-related grade 2–4 AEs. The most common AEs (FPV/r vs EFV) were rash (2% vs 8%), dizziness (0% vs 8%), and hypercholesterolemia (4% vs 4%). Three patients in each group (6%) experienced treatment-related grade 3–4 adverse events (only hypercholesterolemia occurred in >1 patient). Treatment-emergent grade 3–4 laboratory abnormalities emerged in 31% (16/51) of patients taking FPV/r and 24% (12/50) of patients taking EFV; the most common abnormalities involved cholesterol, creatine kinase, and total neutrophils.

**Figure 2:**
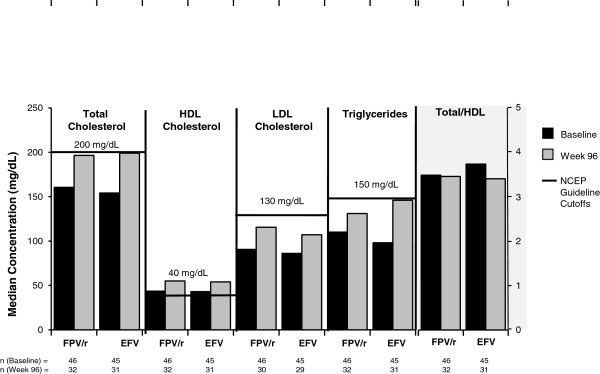
Median fasting lipid concentrations at baseline and at 96 weeks.

Median concentrations of total, LDL, and HDL cholesterol and triglycerides increased over 96 weeks of treatment in both groups (Figure 2), but none of the median values exceeded the maximum concentrations considered within normal limits established by the National Cholesterol Education program (NCEP). At Week 96, median change from baseline (range) in total/HDL cholesterol ratio was -0.03 (-2.38, 2.77) for the FPV/r group and -0.22 (-2.64, 2.04) for the EFV group.

### Cardiovascular biomarkers

At Week 96, plasminogen, sVCAM, d-dimer, and fibrinogen levels decreased significantly from baseline in the EFV group (by 39%, 42%, 39%, and 12%, respectively) while sVCAM and d-dimer decreased significantly from baseline in the FPV/r group (by 48% and 37%, respectively) (Table 3).

**Table 3:** Geometric mean ratios (week 96 to baseline) and 95% confidence intervals

**Biomarker (no. of samples per arm)**	**ABC/3TC + FPV/r**	**ABC/3TC + EFV**
hs-CRP	0.99	1.59
(n = 34, 32)	(0.56, 1.74)	(0.99, 2.56)
Plasminogen	0.73	0.61*
(n = 34, 31)	(0.52, 1.02)	(0.44, 0.85)
sVCAM-1	0.52*	0.58*
(n = 35, 32)	(0.46, 0.58)	(0.53, 0.64)
d-dimer	0.63*	0.61*
(n = 35, 31)	(0.47, 0.84)	(0.48, 0.78)
Interleukin-6	0.77	0.89
(n = 35, 32)	(0.53, 1.14)	(0.58, 1.36)
Fibrinogen	1.02	0.88*
(n = 33, 30)	(0.93, 1.13)	(0.78, 0.99)

* P < 0.05.

**Figure 3:**
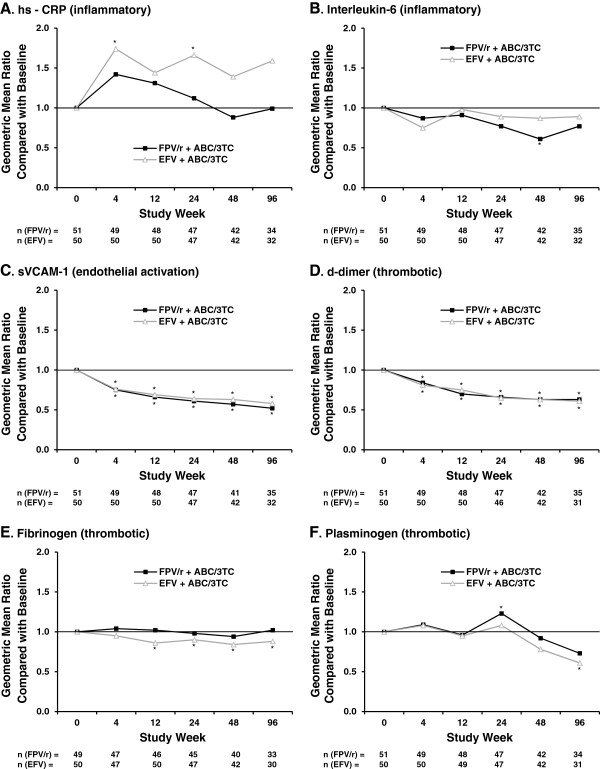
**Change from baseline in cardiovascular biomarkers (expressed as geometric mean ratios) over 96 weeks.** Inflammatory biomarkers: **A** = hs-CRP, **B** = interleukin-6. Endothelial activation biomarker: **C** = sVCAM-1. Thrombotic biomarkers: **D** = d-dimer, **E** = fibrinogen, **F** = plasminogen.

Over 96 weeks of treatment, there was no statistically significant difference between the FPV/r- and EFV-containing groups at any time point for any of the 6 studied biomarkers. However, statistically significant changes were observed in some of the biomarkers when each treatment group was compared with baseline. The inflammatory biomarker hs-CRP (Figure 3A) increased during the first 4 weeks of treatment in both groups, but returned to baseline values in the FPV/r group. In the EFV group, hs-CRP levels remained elevated for the duration of the study, with the increase reaching statistical significance at Weeks 4 and 24. Conversely, levels of the inflammatory biomarker interleukin-6 (Figure 3B) were lower than baseline at all time points in both treatment groups. This difference reached statistical significance only for the FPV/r group at Week 48. Levels of sVCAM-1 (Figure 3C), a biomarker of endothelial activation, and d-dimer (Figure 3D), a thrombotic biomarker, were significantly lower than baseline at all time points for both treatment groups. Fibrinogen (Figure 3E), another thrombotic biomarker, showed no change over time in the FPV/r group but decreased in the EFV group, with the difference reaching statistical significance at Weeks 12, 24, 48, and 96. The final thrombotic biomarker, plasminogen (Figure 3F), was unchanged over the first 12 weeks on therapy in both treatment groups. At Week 24, plasminogen levels increased in both groups, reaching statistical significance for FPV/r. Between Weeks 24 and 96, plasminogen levels decreased in both groups, with the difference reaching statistical significance for EFV at Week 96.

## Discussion

This study prospectively investigated changes in inflammatory, thrombotic, and endothelial activation biomarkers among mostly African-American and Hispanic patients randomized to receive either a boosted protease inhibitor or a non-nucleoside reverse transcriptase inhibitor, each with a backbone of ABC/3TC. Over 96 weeks, except for hs-CRP, biomarker levels generally remained stable or declined overall, with statistically significant declines observed in d-dimer and sVCAM-1 in both groups, and in plasminogen and fibrinogen in the EFV group.

Overall, this study comprising a racially diverse, underrepresented patients, showed no evidence of decreased virologic or immunologic response to either a FPV/r or EFV-based ART with an ABC/3TC backbone, when compared with studies performed in less diverse patient populations. Previous data on race and virologic outcome are conflicting, but several studies [17-22] have documented a decrease in the efficacy of EFV among people of African descent that was not observed here. Few patients (8% and 12%) in this diverse population reached the primary study endpoint in either treatment group, and there was no apparent relationship between the primary endpoint and race, sex, or baseline viral load. Rates of virologic suppression were also similar between the two groups, and patients with screening HIV-1 RNA ≥100,000 c/mL were more likely to have HIV-1 RNA <50 copies/mL at Week 96 than patients with screening HIV-1 RNA <100,000 c/mL because of higher dropout rates among patients in the lower viral load strata in both treatment groups. The higher dropout rates observed for the lower viral load strata may be due to chance given the small sample size of this study. Other randomized clinical trials of treatment-naïve patients have included FPV/r or EFV in combination with ABC/3TC once daily, but, to our knowledge, this study is the first to compare these regimens directly.

There was one fatality in this study. The 49-year old male patient in the FPV/r arm with pre-existing hypercholesterolemia and uncontrolled hypertension had a fatal cardiac arrest after 24 weeks of study and autopsy results confirmed atherosclerotic coronary artery disease with 95% narrowing of the left anterior descending coronary artery. Previous observational studies have linked ritonavir-boosted protease inhibitors and abacavir to the development of myocardial infarctions [23-25]. More recent observational studies and meta-analyses have not shown an association between abacavir use and increased risk of cardiovascular complications [26-28].

HIV treatment guidelines now endorse the use of prospective HLA-B*5701 screening in patients before initiating an ABC-containing regimen to reduce the risk of developing a abacavir hypersensitivity reaction [29]. In the present study, two patients who were of African-American and mixed races, discontinued study after developing symptoms that were thought to be possibly related to abacavir hypersensitivity reaction. Symptoms resolved 7 to 9 days after discontinuation of study regimens. It is noteworthy that in the ARIES trial, which was a large, multicenter study also conducted in the United States, the incidence of clinically suspected abacavir hypersensitivity reaction in HLA-B*5701–negative patients during the first 30 weeks was 0.8% [30]. However, when these patients underwent abacavir skin-patch testing (a research tool not for clinical use) at least 6 weeks after symptom resolution, 0% had a positive skin-patch test, indicating that they did not have immunologically confirmed abacavir hypersensitivity reaction.

In this study, which evaluated exclusively patients from racial and ethnic minority populations, higher levels of hs-CRP were observed in the EFV + ABC/3TC group compared with baseline, although the difference was statistically significant only at Weeks 4 and 24, while short-term, non-significant increases from baseline were observed for hs-CRP levels in the FPV/r + ABC/3TC group. Our results are generally consistent with previous studies which have shown hs-CRP to be unchanged or increased with ART initiation. In ACTG5095, a double-blind comparison of zidovudine/3TC + EFV and zidovudine/3TC/ABC + EFV, a post-hoc analysis of 196 patients with HIV-1 RNA <50 copies/mL at Weeks 24 and 96 found that hs-CRP was not statistically different between baseline and Week 96 [11]. Similar results were observed in a post-hoc analysis of HEAT, a study comparing ABC/3TC and TDF/FTC in combination with ritonavir-boosted lopinavir [12]. At Week 96, hs-CRP decreased modestly from baseline in both treatment groups with no statistical difference observed between the groups; no data were reported regarding the statistical significance of the changes relative to baseline. Finally, ACTG A5224s which evaluated biomarkers in 244 patients with available stored plasma samples, variability in hs-CRP response was noted over time and between treatment groups [14]. Consistent with our study, hs-CRP was significantly higher than baseline at Weeks 24 and 96 (the only time points measured) in the EFV arm but hs-CRP levels did not vary significantly from baseline in the boosted protease inhibitor arm.

A number of factors may explain the variability in hs-CRP response. One reason proposed may be due to hepatocyte dysfunction attributed to HIV infection [14]. Our findings may also reflect genetic or societal risk factors associated with the study population. In contrast to some of the previously mentioned studies which have some minority representation, this study was fully recruited with a diverse patient population. Furthermore, the health risk factors observed in this cohort may have been greater since half of the enrolled subjects were current or former smokers with a median smoking history of 10 years, and a considerable proportion of the enrolled subjects had health issues including hypertension, and hypercholesterolemia. Given these considerations, hs-CRP may not be a reliable biomarker of inflammation in HIV-infected individuals as it is in HIV-negative individuals [31].

Decreases in IL-6 and sVCAM-1 in this population of racial and ethnic minorities initiating ARV appear generally consistent with results noted in previous randomized clinical trials of ART-naïve patients. In this study, values for IL-6 decreased from baseline in both treatment groups, but the change never reached statistical significance by Week 96. In the HEAT study, IL-6 also decreased between baseline and Weeks 48 and 96, with similar declines in both treatment groups [12]. In ACTG A5224s, IL-6 also declined over time, and the changes from baseline to Week 96 were statistically significant in all 4 treatment groups [14]. Importantly, increased IL-6 was shown to be associated with a higher mortality and more cardiovascular events as seen in the SMART study [32]. In our study, sVCAM-1 significantly decreased from baseline at all time points in both treatment groups. Results from HEAT were similar, with a 50% decline in sVCAM-1 levels in both treatment groups between baseline and Week 96 (no p-values reported) [12]. Similarly, in ACTG A5224s, sVCAM-1 decreased significantly from baseline to Week 96 in all treatment groups [14].

None of the three randomized trials in ARV-naïve patients (ACTG5095, HEAT, ACTG5224s) investigated changes in d-dimer, fibrinogen, or plasminogen. However, data from observational cohorts support similar trends for all 3 biomarkers compared with changes observed in this study [9,13,32-35]. In particular, the SMART study found significantly lower d-dimer levels in patients taking combination ART for the previous 12 months compared with patients who were off therapy [32]. Importantly, increases in d-dimer have been shown to be associated with the higher mortality and cardiovascular events reported in the SMART study [32].

A key limitation of this study is that the clinical significance of the biomarker findings is unclear, and the study did not assess the association between the biomarkers and more clinical evidence of endothelial dysfunction such as changes of flow-mediated dilation [36,37]. Other limitations of this study include the open-label design and modest sample size. Strengths of this study include its randomized design, the 96-week follow-up period of the study, and real-time analysis of the biomarkers in a racially diverse HIV-infected population with a sizable proportion of female study participants.

## Conclusions

In this study of primarily African-Americans and Hispanics patients typically underrepresented in clinical trials, ART initiation with either FPV/r or EFV, in combination with ABC/3TC resulted in high levels of virologic suppression in subjects who continued in the study through 96 weeks, with consistent and favorable decreases in thrombotic activity, as reflected by d-dimer changes, and in endothelial activation, as reflected by sVCAM-1 changes. Except for hs-CRP, concentrations of other biomarkers generally remained stable or declined overall, with statistically significant declines observed in plasminogen and fibrinogen in the EFV group. Our study adds to the emerging data that some biomarkers are decreased with ARV therapy and control of HIV viremia. Additional studies are needed to further clarify the clinical significance of these findings and further elucidate whether biomarkers can be used for risk stratification in identifying patients that most benefit from aggressive management of CVD risk.

## Competing interests

Princy Kumar, MD, has received consulting fees from Bristol-Myers Squibb. She has served on speakers’ bureaus for Boehringer Ingelheim, GlaxoSmithKline, Tibotec Pharmaceuticals, and ViiV HealthCare and has received research funding from GlaxoSmithKline, Merck, and Tibotec Pharmaceuticals.

Edwin DeJesus, MD, has received consulting fees from and served on speakers’ bureaus for Gilead Sciences, Merck, and Tibotec Pharmaceuticals. He has received research funding from Abbott Laboratories, Achillion, Avexa, Boehringer Ingelheim, Bristol-Myers Squibb, Gilead Sciences, GlaxoSmithKline, Hoffman LaRoche Laboratories, Merck, Pfizer, Schering Plough, Taimed, Tobira, Tibotec Pharmaceuticals, and Vertex Pharmaceuticals.

Gregory Huhn, MD, has received consulting fees from GlaxoSmithKline, Gilead Sciences, Vertex Pharmaceuticals, and Tibotec Pharmaceuticals. He has served on speakers’ bureaus for Genentech, GlaxoSmithKline, Gilead Sciences, Merck, Novartis, Sanofi Pasteur, Tibotec Pharmaceuticals, and ViiV HealthCare and has received research funding from GlaxoSmithKline, Gilead Sciences, Merck, Tibotec Pharmaceuticals, and Vertex Pharmaceuticals.

Louis Sloan, MD, has received consulting fees from, served on speakers’ bureaus for, and received research funding from ViiV Healthcare.

Catherine Butkus Small, MD, has received consulting fees from Schering-Plough. She has received research funding from Abbott Laboratories, GlaxoSmithKline, Pfizer, Schering-Plough, and ViiV Healthcare.

Howard Edelstein, MD, has received consulting fees from Gilead Sciences. He has received research funding from Bristol-Myers Squibb, Gilead Sciences, Pfizer, and ViiV Healthcare.

Franco Felizarta, MD, has received consulting fees from Merck and Gilead Sciences. He has served on speakers’ bureaus for and has received research funding from Boehringer Ingelheim, GlaxoSmithKline, Merck, Pfizer, Tibotec Pharmaceuticals, and ViiV Healthcare.

Ritche Hao, MD, has received consulting fees from Gilead Sciences. He has received research funding from GlaxoSmithKline, Gilead Sciences, Merck, Napo Pharmaceuticals, Merck, Tibotec Pharmaceuticals, and ViiV Healthcare.

Lisa Ross, Britt Stancil, Keith Pappa, PharmD, and Belinda Ha, PhD, are employees of GlaxoSmithKline and ViiV Healthcare.

## Authors’ contributions

PK contributed to the design of this study. PK, ED, GH, LS, CS, HE, FF, and RH enrolled patients in this study. PK and BH were involved in drafting this manuscript, LR and BS contributed to data analysis, and all authors provided critical review of the manuscript and approved it for publication.

## Pre-publication history

The pre-publication history for this paper can be accessed here:

http://www.biomedcentral.com/1471-2334/13/269/prepub
